# Use of face masks to limit the spread of the COVID-19 among western Ugandans: Knowledge, attitude and practices

**DOI:** 10.1371/journal.pone.0248706

**Published:** 2021-03-24

**Authors:** Franck Katembo Sikakulya, Robinson Ssebuufu, Simon Binezero Mambo, Theophilus Pius, Annet Kabanyoro, Elizabeth Kamahoro, Yusuf Mulumba, Jean Kakule Muhongya, Patrick Kyamanywa

**Affiliations:** 1 Faculty of Clinical Medicine and Dentistry, Department of Surgery, Kampala International University Western Campus, Ishaka-Bushenyi, Uganda; 2 Faculty of Medicine, Université Catholique du Graben, Butembo, Democratic Republic of the Congo; 3 Youth Alliance for Reproductive Health, Goma, Democratic Republic of the Congo; 4 Department of Medical Laboratory Sciences, Kampala International University Western Campus, Ishaka-Bushenyi, Uganda; 5 School of Nursing Sciences, Kampala International University Western Campus, Ishaka-Bushenyi, Uganda; 6 Biostatistics, Cancer Institute, Makerere University, Kampala, Uganda; 7 Allied Health Sciences Department, Kampala International University Western Campus, Ishaka-Bushenyi, Uganda; IRCCS Neuromed, CUAMM, ITALY

## Abstract

**Background:**

The world is grappling with an ever-changing COVID-19 pandemic using preventive measures such as personal hygiene, face masks, restrictions on travel and gatherings in communities, in addition to a race to find a vaccine. The purpose of this study was to evaluate the knowledge, attitudes and practices of the western Uganda community on the proper use of face masks to mitigate the spread of COVID-19.

**Methods:**

A cross-sectional study using a structured questionnaire was carried out from 1^st^ July to 10^th^ July 2020 among western Ugandans of consent age of 18 years and above. Data was analysed using Stata version 14.2.

**Results:**

Among the respondents (n = 1114), the mean age was 30.7 (SD 11.1), 51% were males, 53.9% married and 43% had attained secondary education. Most participants (60.1%, n = 670) had satisfactory knowledge on the use of face masks and participants at a tertiary education level [AOR 2.6 (95% CI: 1.42–4.67; p = 0.002)] were likely to have satisfactory knowledge than participants who had not education. On attitude, most respondents (69.4%) were confident enough to correctly put on a face mask; 83.4% believed that a face mask can protect against COVID-19 and 75.9% of respondents had never shared their face mask. The majority of respondents (95.2%) agreed wearing face masks in public places was important to protect themselves against COVID-19; 60.3% reported washing their hands before wearing and after removing the face mask. Unfortunately, 51.5% reported removing the face mask if they needed to talk to someone.

**Conclusion:**

Despite the satisfactory knowledge, good attitude and practices, there is still much more to be done in terms of knowledge, attitude and practices among participants. Government, non-governmental organizations and civil society should improve sensitization of populations on how to behave with face masks while talking to avoid the spread of the COVID-19 among western Ugandans.

## 1. Introduction

Coronavirus Disease 2019 (COVID-19) is a respiratory disease caused by the SARS-CoV-2 virus and on March 11, 2020, the World Health Organization (WHO) declared COVID-19 a pandemic [1]. According to the WHO, as of 5^th^ March 2021, a total of 116,614,624 cases of COVID-19 had been confirmed worldwide (3,971,496 confirmed in Africa). Information suggests that the two main routes of transmission of the COVID-19 virus are respiratory droplets and contact [2].

It is known that the novel COVID-19 has an incubation period of 2 to 14days during which all infected patients, asymptomatic or with mild symptoms, transmit the disease to a non-infected person [1] and this poses a challenge for early isolation and containment, of community transmission [3].

In order to minimize the risk, the public is required to follow accepted infection control practices [4] and these include community-based measures such as self-isolation, use of alcohol-based hand sanitizer or hand-washing with soap, restriction of movements with lock down measures, sanitization of surfaces and use of non-medical cloth mask or face covering [5, 6]. During the on-going COVID-19 pandemic, recommendations and common practices regarding face mask use by the general public have varied greatly [7, 8].

There has been much debate globally and locally about whether members of the general public should be advised to wear face-masks during the COVID-19 pandemic [4, 9, 10]. However as the epidemic rages on, support for the wide use of cloth face-masks, including people who are not ill, is growing [4]. The main benefit of everyone wearing a face-mask is to reduce the amount of Coronavirus (or Influenza virus) being released to the environment by those with the infection thereby reducing its spread through droplets [4]. There are different types of face masks in use by the community and these include N95, N100, N99, surgical mask and cloth masks [11].

A study in the US provides evidence that use of face masks in public resulted in a greater decline in daily COVID-19 growth rates in states that required face mask use compared to states that did not issue such mandates [7]. The WHO states that incorrect use and disposal of face masks may actually increase the rate of transmission [4]. Studies on knowledge and practices about measures to prevent the spread of the COVID-19 pandemic have reported a non-linear relationship between the knowledge and practice of using face masks to prevent the spread of COVID-19 among different categories of participants [8, 12, 13].

The government of Uganda has put in place measures to be observed by the public to avoid the spread of COVID-19 in the communities but studies to evaluate whether the communities are following these measures reported a gap among different categories of Ugandans [14–16]. Given that use of face masks is one of the measures to prevent the spread of COVID-19 among communities [17] and there is no published study yet evaluating the knowledge, attitude and practices of use of face mask among Ugandans, it was imperative to conduct a study on knowledge, attitude and practices of Ugandans about the use of face masks to prevent the spread of COVID-19. Therefore, the aim of this study was to evaluate the knowledge, attitudes and practices on proper use of face masks to limit the spread of COVID-19 among Ugandans located in four districts in western Uganda.

## 2. Methods

### 2.1 Study design

This was a face-to-face community based cross-sectional study conducted in four districts of western Uganda. Western Uganda and in particular the four districts were conveniently chosen given the existing collaboration of the University with the population in this area and the low level of community transmission of COVID-19 at the time of the research.

## 2.2 Study participants, sample size and sampling

According to the Uganda Bureau of Statistics the western region of Uganda (UBOS) has a total of 58 districts and an estimated population of 10,577,900 with 5,225,800 (49.4%) males and 5,352,100 (50.6%) females [18]. Four districts of Kabarole (population of 469,236), Hoima (population of 572,986), Kasese (population of 694,992) and Bushenyi (population of 234,440) were chosen for the study [18]. All Ugandans located in the four districts and aged 18years and above regardless of gender, education level, marital status, religion and occupation were considered eligible for the study.

To calculate the sample size, we hypothesized that at a 99.9% confidence interval, 50% of the respondents would have a satisfactory knowledge level on the use of face masks to limit the spread of the COVID-19. Using the Open-Source Epidemiologic Statistics for Public Health (OpenEpi), v.3.01 (Dean AG, Sullivan KM, Soe MM. Open-Epi: www.OpenEpi.com, updated April 06, 2013), a minimum sample size of 1083 participants was needed in the four districts. By adding 20 percent contingence for non-compliance to the data collection tool, a total sample size of 1300 participants was targeted. Four teams were sent out to the selected districts to recruit and dispense the study tool to the highest number of participants wearing face mask in the given time frame (1^st^ July to 10^th^ July 2020) in accordance with the principle of ‘the larger the sample, the greater the statistical power [19], keeping them blind of the required sample size. The teams were called to stop recruitment when the data collection period time was reached. At the end of the data collection period, 1114 participants (224 in Kabarole, 274 in Hoima, 240 in Kasese and 376 in Bushenyi) had responded to the study tool from the four teams in the four districts. Probability sampling was not feasible in this study because a sampling frame was unavailable. Despite using a nonprobability sampling method, quota sampling increases the representativeness of the sample. A quota of 325 participants was given for each district from the 1300 targeted sample size. This sample was divided according to the preselected four districts preselected and the quota was distributed equally to gender participants with a ratio of male to female of 1:1. In addition, purposive sampling technique was used targeting participants of consent age of 18 years and above with masks.

### 2.3 Data collection and instrument

The study was conducted for a period of ten days from 1^st^ July to 10^th^ July 2020 and participants were asked to respond to a structured questionnaire, pre-validated by two independent reviewers and piloted in Bushenyi district of western Uganda whose responses were not included in the study.

The study tool was designed as a multiple-choice questionnaire with correct and wrong answers to be chosen by the participants during the interview. The questionnaire was composed of 21 items developed based on WHO requirements for face mask use KAP (knowledge, attitudes and practices) [17], focused on several key constructs: Six questions related to socio-demographics characteristics (age, gender, occupation, educational level, marital status and residence); Ten questions (10) knowledge focused on disease spread, symptoms, and how to limit infection by using face masks during the COVID-19 pandemic (Table 2); Three questions focused on behaviour and attitude to wear face masks and three questions on practices on the use of face masks to limit the spread of COVID-19. Knowledge score was categorised into satisfactory for participants who had more than 50% of the correct answers and unsatisfactory for those who had less than 50% of the correct answers.

A face-to-face interview was conducted in an open area, observing measures to prevent the spread of COVID-19 among participants in accordance with the guidelines by the Uganda National Council for Science and Technology (UNCST) (http://www.uncst.go.ug/). A plain language consent explanation was read to the prospective participant by the researcher before the participant agreed to respond to questions. Participants were asked which language they preferred to use for the interview and the appropriate version of the tool was then used.

### 2.4 Data processing and analysis plan

The raw data was cleaned and entered into Microsoft excel and exported into STATA version 14.2 which was used for statistical analysis (StataCorp, College Station, Texas, USA).

Univariate analysis of all socio-demographic characteristics of the participants was done and presented as frequencies and percentages for categorical variables, and median for skewed data with standard deviation (SD) and interquartile range (IQR) respectively for continuous variables.

To assess knowledge of participants, a numeric scoring pattern was used, and outcome variables of knowledge were computed. These outcome variables were further categorized as binary (satisfactory or unsatisfactory) based on cut-off (mean score) marks (Table 3). Participants receiving scores greater than the mean score for knowledge [8.1(2.13)] were deemed to have satisfactory knowledge and vice versa.

Chi-square test was used for association between socio-demographic characteristics and knowledge (satisfactory vs unsatisfactory), attitude and practices at a 95% confidence interval with significant variables (p < 0.05) and subjected to multiple logistic regression for knowledge (Satisfactory vs Unsatisfactory) with socio-demographic characteristics.

### 2.5 Ethical considerations

Ethical clearance for the survey was obtained from the Institutional Research Ethical Committee of Kampala International University in Uganda (UG-REC-023/201914) and informed consent of respondents before enrolling them voluntarily in the study. Ethics issues such as privacy and confidentiality of the respondents were ensured. The potential study benefits to Ugandans were to explore, understand and document the knowledge, attitude and practices towards the use of face masks to limit the spread of the COVID-19 among western Ugandans so as to inform and enhance risk communication to mitigate the risk for contracting the disease through improper use of face masks. With data collected by face-to-face interview, researchers were susceptible to contracting COVID-19 infection. However, to overcome this, all research assistants underwent a training on infection prevention control and how to observe standard operating procedures by putting on personal protective equipment during data collection in accordance with the guidelines set by the Uganda National Council for Science and Technology (UNCST) and the Ministry of Health. Following the training, all the research assistants were each provided with a box of 30 disposable surgical face masks, alcohol hand sanitizer and face-shields for use during data collection.

## 3. Results

### 3.1 Socio-demographic characteristics of participants

A total of 1114 Ugandans from Kabarole (224), Hoima (274), Kasese (240) and Bushenyi (376) participated in the study. As shown in Table 1, most participants (64.2%, n = 715/1114) were aged between 18–30 years with a median age of 28 (SD 13); married (53.9%, n = 600/1114). The majority of the participants had secondary level education (43.0%, n = 479/1114), and were from urban settings 55.1% (n = 614/1114).

**Table 1:** Socio-demographic characteristics of the respondents.

Variable	Frequency (Percent)
Sample size	1114 (100.0)
Age group in years	
18 to 27	529 (47.5)
28 to 37	340 (30.5)
38 to 47	153 (13.7)
48 and above	92 (8.3)
Gender	
Male	568 (51.0)
Female	546 (49.0)
Marital status	
Single	449 (40.3)
Married	600 (53.9)
Cohabiting	65 (5.8)
Education level	
No formal education	73 (6.6)
Primary	210 (18.9)
Secondary	479 (43.0)
Tertiary	352 (31.6)
Occupation	
Student	253 (22.7)
Business	243 (21.8)
Farmer	155 (13.9)
Health worker	120 (10.8)
Household-wife	66 (5.9)
Civilian servant	184 (16.5)
Unemployed	93 (8.3)
Residence	
Rural	500 (44.9)
Urban	614 (55.1)

### 3.2 Description of knowledge questions and score obtained by the participants (Tables 2, 3)

**Table 2:** Questions on knowledge of western Ugandans towards the use of face mask to limit the spread of COVID-19.

Questions	Yes (%)	No (%)	I Don’t know (%)
What is the mode of transmission of covid-19? (Multiple choice)	-	-	-
Contact routes (Correct answer)	441 (39.6)	673 (60.4)	0 (0.0)
Respiratory droplets (Correct answer)	634 (56.9)	480 (43.1)	0 (0.0)
Don’t know	77 (6.9)	1037 (93.1)	0 (0.0)
Wearing a face mask frequently is one of the ways of reducing transmission of COVID-19	1006 (90.3)	74 (6.6)	34 (3.1)
It is believed that face masks actually protect against COVID-19	962 (86.4)	105 (9.4)	47 (4.2)
Cloth face mask is effective as a regular surgical face mask or N95 on limiting the spread of COVID-19	739 (66.3)	256 (23)	119 (10.7)
It is necessary to wear a face mask when you don’t have COVID-19	962 (86.4)	123 (11)	29 (2.6)
Face masks protect someone from getting COVID-19	963 (86.4)	110 (9.9)	41 (3.7)
Infected individuals reduce the risk of spreading the COVID-19 to others by wearing face masks	968 (86.9)	118 (10.6)	28 (2.5)
Can widespread use of face masks in a population facilitate the control of COVID-19?	957 (85.9)	115 (10.3)	42 (3.8)
For proper wearing, the face masks should cover the nose, mouth, and chin	506 (45.4)	608 (54.6)	0 (0.0)
To which category of people are face masks useful? (Multiple choice)	-	-	-
Everyone in public area (Correct answer)	975 (87.5)	128 (11.5)	11 (1.0)
Six years and above (Correct answer)	34 (3.1)	1080 (96.9)	0 (0.0)
Health workers only	62 (5.6)	1052 (94.4)	0 (0.0)
Truck drivers only	26 (2.3)	1088 (97.7)	0 (0.0)
Patients with COVID-19 only	40 (3.6)	1074 (96.4)	0 (0.0)
None	11 (1)	1103 (99.0	0 (0.0)

**Table 3:** Description of knowledge score obtained by participants (n = 1114).

Outcome variable		Knowledge
**Scores**	Maximum attainable score	12
	Minimum	0
Maximum		10
Mean (SD)		8.1(2.13)
Satisfactory n (%)		670 (60.1)
Unsatisfactory n (%)		444 (39.9)

The participants were able to respond to the questions related to knowledge towards the use of face mask to limit the spread of COVID-19 within the community. Although, the response rate varied across questions as indicated in Table 2 below.

The mean knowledge score was 8.1±2.13, from a maximum attainable score of 12 (Table 3). Most participants (60.1%, n = 670/1114) had satisfactory knowledge on the use of face masks to limit the spread of COVID-19 among western Ugandans.

### 3.3 Socio-demographic factors influence knowledge of participants on the use of face masks to limit the spread of COVID-19

As expected, participants at a tertiary education level were 1.5 times (95% CI: 1.01–2.19; p = 0.047) more likely to have satisfactory knowledge of the use of face mask to limit the spread of COVID-19 than participants who had not education **(Table 4).**

**Table 4:** Socio-demographic factors influence knowledge of participants about the use of face masks to limit the spread of COVID-19.

Variable	Total (%)	Satisfactory	Unsatisfactory (%)	P-Value (x^2^)	AOR (95%CI)	P-Value
**Sample size**	**1114 (100)**	**670 (60.1)**	**444 (39.9)**			
**Age group in years**				**0.125**		
18 to 27	715 (100)	432 (60.4)	283 (39.6)		1	.
28 to 37	335 (100)	202 (60.3)	133 (39.7)		1.1 (0.85–1.32)	0.617
38 and 47	64 (100)	36 (56.3)	28 (43.8)		1.1 (0.85–1.46)	0.445
48 and above					1 (0.69–1.36)	0.861
**Gender**				**0.435**		
Male	568 (100)	348 (61.3)	220 (38.7)		1	
Female	546 (100)	322 (59)	224 (41)		1 (0.83–1.15)	0.791
**Marital status**				**0.049**		
Single	449 (100)	256 (57)	193 (43)		1	.
Married	600 (100)	380 (63.3)	220 (36.7)		1.1 (0.92–1.42)	0.241
Cohabiting	65 (100)	34 (52.3)	31 (47.7)		1 (0.67–1.39)	0.854
**Education level**				**0.008**		
No formal education	73 (100)	34 (46.6)	39 (53.4)		1	.
Primary	210 (100)	117 (55.7)	93 (44.3)		1.2 (0.80–1.74)	0.405
Secondary	479 (100)	288 (60.1)	191 (39.9)		1.3 (0.91–1.94)	0.144
Tertiary	352 (100)	231 (65.6)	121 (34.4)		1.5 (1.01–2.19)	0.047
**Occupation**				**0.098**		
Student	253 (100)	156 (61.7)	97 (38.3)		1	.
Business	243 (100)	154 (63.4)	89 (36.6)		0.9 (0.71–1.21)	0.593
Farmer	155 (100)	103 (66.5)	52 (33.5)		1.1 (0.76–1.44)	0.772
Health worker	120 (100)	72 (60)	48 (40)		0.8 (0.59–1.12)	0.21
Housewife	66 (100)	35 (53)	31 (47)		0.8 (0.51–1.16)	0.21
Civilian servant	184 (100)	104 (56.5)	80 (43.5)		0.8 (0.60–1.06)	0.125
Unemployed	93 (100)	46 (49.5)	47 (50.5)		0.8 (0.56–1.14)	0.221
**Residence**				**0.187**		
Rural	500 (100)	290 (58)	210 (42)		1	
Urban	614 (100)	380 (61.9)	234 (38.1)		1 (0.88–1.22)	0.671

**AOR:** Adjusted Odds Ratio

### 3.4 Attitude towards the use of face masks among participants

**Fig 1:**
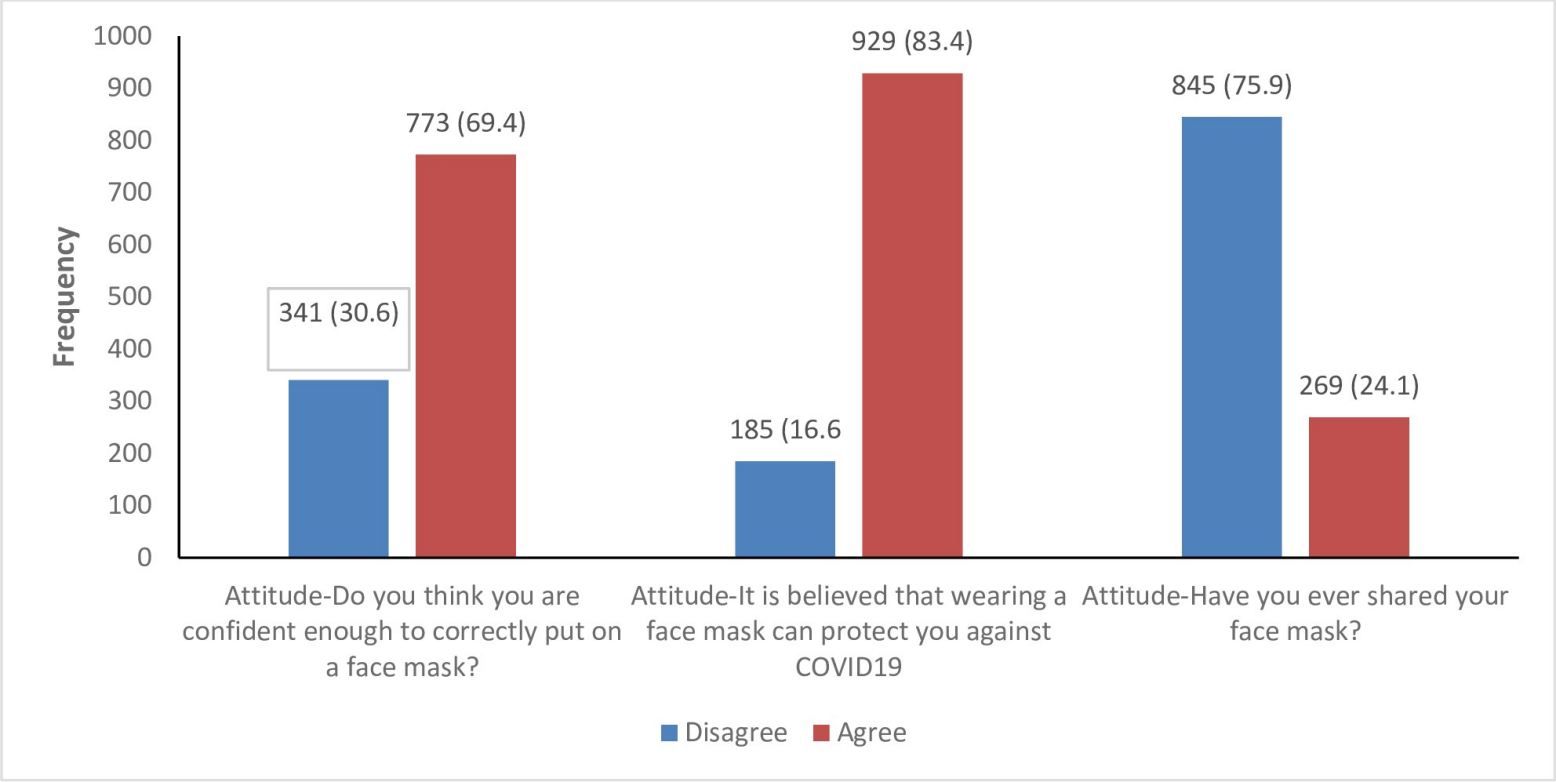
Attitude towards the use of face masks to limit the spread of COVID-19 among western Ugandans.

Most participants (69.4%) were confident enough to correctly put on a face mask; 83.4% believed that a face mask can protect against COVID-19 and 75.9% disagreed to ever share their face mask (**Fig 1**).

### 3.5 Attitude and socio-demographic characteristics of participants

As shown in Table 5, the attitude of being confident enough to correctly put on a face mask differed across education levels and occupation of the participants (P<0.05); the attitude to believe that a face mask can protect against COVID-19 varied across age group, marital status and residence (p<0.005) and the attitude about sharing the face mask varied across the marital status (p<0.005).

**Table 5:** Attitude and socio-demographic characteristics of participants on the use of face masks to limit the spread of COVID-19.

Variable			A1		A2			A3		
	Total (%)	Agree (%)	Disagree (%)	P-Value	Agree (%)	Disagree (%)	P-Value	Yes (%)	No (%)	P-Value
Sample size	1114 (100)	773 (69.4)	341 (30.6)		929 (83.4)	185 (16.6)		269 (24.1)	845 (75.9)	
Age group in years				0.770			**0.009**			0.057
18 to 27	529 (100.0)	372 (70.3)	157 (29.7)		423 (80.0)	106 (20.0)		138 (26.1)	391 (73.9)	
28 to 37	340 (100.0)	237 (69.7)	103 (30.3)		298 (87.6)	42 (12.4)		82 (24.1)	258 (75.9)	
38 and 47	153 (100.0)	104 (68.0)	49 (32.0)		134 (87.6)	19 (12.4)		24 (15.7)	129 (84.3)	
48 and above	92 (100.0)	60 (65.2)	32 (34.8)		74 (80.4)	18 (19.6)		25 (27.2)	67 (72.8)	
Gender				0.372			0.238			0.200
Male	568 (100)	401 (70.6)	167 (29.4)		481 (84.7)	87 (15.3)		128 (22.5)	440 (77.5)	
Female	546 (100)	372 (68.1)	174 (31.9)		448 (82.1)	98 (17.9)		141 (25.8)	405 (74.2)	
Marital status				0.919			**0.001**			**0.003**
Single	449 (100)	310 (69)	139 (31)		369 (82.2)	80 (17.8)		128 (28.5)	321 (71.5)	
Married	600 (100)	419 (69.8)	181 (30.2)		518 (86.3)	82 (13.7)		121 (20.2)	479 (79.8)	
Cohabiting	65 (100)	44 (67.7)	21 (32.3)		42 (64.6)	23 (35.4)		20 (30.8)	45 (69.2)	
Education level				**0.001**			0.075			0.911
No formal education	73 (100)	39 (53.4)	34 (46.6)		54 (74)	19 (26)		16 (21.9)	57 (78.1)	
Primary	210 (100)	134 (63.8)	76 (36.2)		170 (81)	40 (19)		53 (25.2)	157 (74.8)	
Secondary	479 (100)	338 (70.6)	141 (29.4)		405 (84.6)	74 (15.4)		118 (24.6)	361 (75.4)	
Tertiary	352 (100)	262 (74.4)	90 (25.6)		300 (85.2)	52 (14.8)		82 (23.3)	270 (76.7)	
Occupation				**0.002**			0.077			0.664
Student	253 (100)	187 (73.9)	66 (26.1)		215 (85)	38 (15)		66 (26.1)	187 (73.9)	
Business	243 (100)	169 (69.5)	74 (30.5)		208 (85.6)	35 (14.4)		65 (26.7)	178 (73.3)	
Farmer	155 (100)	105 (67.7)	50 (32.3)		132 (85.2)	23 (14.8)		39 (25.2)	116 (74.8)	
Health worker	120 (100)	97 (80.8)	23 (19.2)		101 (84.2)	19 (15.8)		23 (19.2)	97 (80.8)	
Housewife	66 (100)	41 (62.1)	25 (37.9)		58 (87.9)	8 (12.1)		15 (22.7)	51 (77.3)	
Professional	184 (100)	122 (66.3)	62 (33.7)		147 (79.9)	37 (20.1)		39 (21.2)	145 (78.8)	
Unemployed	93 (100)	52 (55.9)	41 (44.1)		68 (73.1)	25 (26.9)		22 (23.7)	71 (76.3)	
Residence				0.891			**0.001**			0.192
Rural	500 (100)	348 (69.6)	152 (30.4)		388 (77.6)	112 (22.4)		130 (26)	370 (74)	
Urban	614 (100)	425 (69.2)	189 (30.8)		541 (88.1)	73 (11.9)		139 (22.6)	475 (77.4)	

**A1:** Attitude-Do you think you are confident enough to correctly put on a face mask?

**A2:** Attitude-It is believed that wearing a face mask can protect you against COVID19

**A3:** Attitude-Have you ever shared your face mask?

### 3.6 Practices towards the use of face masks among participants

**Fig 2:**
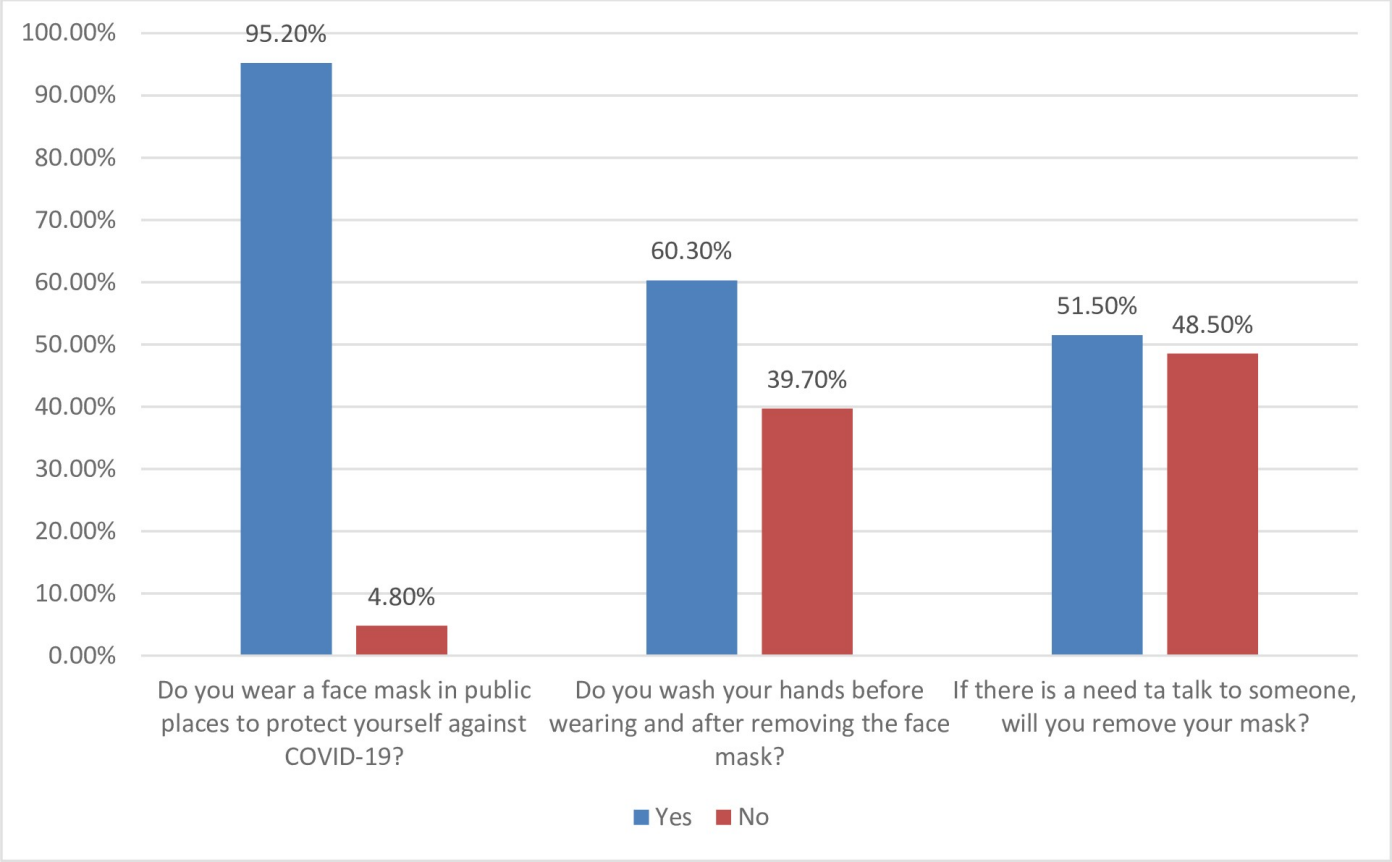
Practices towards the use of face masks to limit the spread of COVID-19 among western Ugandans.

Most participants (95.2%) agreed to wearing a face mask in public places to protect themselves against COVID-19; 60.3% washed their hands before wearing and after removing the face mask but 51.5% agreed to removing the face mask if there is a need to talk to someone (Fig 2).

### 3.7 Practices and socio-demographic of participants

The practices of wearing face masks in public places differed across education levels and occupation of participants (P<0.05); the practices about washing hands before wearing and after removing the face mask varied across occupation and residence (p<0.005). Removing a face mask if there is a need to talk to someone also varied across educational level (p<0.05) **(Table 6).**

**Table 6:** Practices and socio-demographic of participants on the use of face masks to limit the spread of COVID-19.

Variable			P1		P2			P3		
	Total (%)	Yes (%)	No (%)	P-Value	Yes (%)	No (%)	P-Value	No (%)	Yes (%)	P-Value
Sample size	1114 (100)	1060 (95.2)	54 (4.8)		672 (60.3)	442 (39.7)	** **	574 (51.5)	540 (48.5)	
Age group in years				0.288			0.278			0.054
18 to 27	529 (100.0)	504 (95.3)	25 (4.7)		329 (62.2)	200 (37.8)		270 (51.0)	259 (49.0)	
28 to 37	340 (100.0)	328 (96.5)	12 (3.5)		192 (56.5)	148 (43.5)		168 (49.4)	172 (50.6)	
38 and 47	153 (100.0)	143 (93.5)	10 (6.5)		91 (59.5)	62 (40.5)		59 (38.6)	94 (61.4)	
48 and above	92 (100.0)	85 (92.4)	7 (7.6)		60 (65.2)	32 (34.8)		43 (46.7)	49 (53.3)	
**Gender**				0.333			0.867			0.873
Male	568 (100)	537 (94.5)	31 (5.5)		344 (60.6)	224 (39.4)		294 (51.8)	274 (48.2)	
Female	546 (100)	523 (95.8)	23 (4.2)		328 (60.1)	218 (39.9)		280 (51.3)	266 (48.7)	
**Marital status**				0.463			0.608			**0.001**
Single	449 (100)	430 (95.8)	19 (4.2)		275 (61.2)	174 (38.8)		211 (47)	238 (53)	
Married	600 (100)	570 (95)	30 (5)		355 (59.2)	245 (40.8)		338 (56.3)	262 (43.7)	
Cohabiting	65 (100)	60 (92.3)	5 (7.7)		42 (64.6)	23 (35.4)		25 (38.5)	40 (61.5)	
**Education level**				**0.001**			0.711			**0.001**
No formal education	73 (100)	64 (87.7)	9 (12.3)		42 (57.5)	31 (42.5)		38 (52.1)	35 (47.9)	
Primary	210 (100)	194 (92.4)	16 (7.6)		122 (58.1)	88 (41.9)		89 (42.4)	121 (57.6)	
Secondary	479 (100)	458 (95.6)	21 (4.4)		288 (60.1)	191 (39.9)		235 (49.1)	244 (50.9)	
Tertiary	352 (100)	344 (97.7)	8 (2.3)		220 (62.5)	132 (37.5)		212 (60.2)	140 (39.8)	
**Occupation**				**0.026**			**0.001**			**0.001**
Student	253 (100)	246 (97.2)	7 (2.8)		154 (60.9)	99 (39.1)		122 (48.2)	131 (51.8)	
Business	243 (100)	227 (93.4)	16 (6.6)		117 (48.1)	126 (51.9)		124 (51)	119 (49)	
Farmer	155 (100)	140 (90.3)	15 (9.7)		96 (61.9)	59 (38.1)		73 (47.1)	82 (52.9)	
Health worker	120 (100)	117 (97.5)	3 (2.5)		93 (77.5)	27 (22.5)		83 (69.2)	37 (30.8)	
Housewife	66 (100)	64 (97)	2 (3)		38 (57.6)	28 (42.4)		32 (48.5)	34 (51.5)	
Professional	184 (100)	176 (95.7)	8 (4.3)		116 (63)	68 (37)		101 (54.9)	83 (45.1)	
Unemployed	93 (100)	90 (96.8)	3 (3.2)		58 (62.4)	35 (37.6)		39 (41.9)	54 (58.1)	
**Residence**				0.438			**0.006**			**0.003**
Rural	500 (100)	473 (94.6)	27 (5.4)		324 (64.8)	176 (35.2)		233 (46.6)	267 (53.4)	
Urban	614 (100)	587 (95.6)	27 (4.4)		348 (56.7)	266 (43.3)		341 (55.5)	273 (44.5)	

**P1:** Do you wear a face mask in public places to protect yourself against COVID-19?

**P2:** Do you wash your hands before wearing and after removing the face mask?

**P3:** If there is a need to talk to someone, will you remove your mask?

## 4. Discussion

It has been confirmed that patients with mild or no symptoms at the pre-symptomatic and early stages of infection can contribute to the spread of COVID-19 [11]. A face mask may help to reduce the spread of infection in the community by minimising the excretion of respiratory droplets from infected individuals but studies are still controversial on the type of mask to be used in this current COVID-19 pandemic [11].

A study on KAP can contribute to a better understanding of the current situation, obstacles, and solutions for policy formation by the government through the Ministry of Health.

In this study, most participants (60.1%) had satisfactory knowledge on the use of face masks to limit the spread of COVID-19 and this knowledge score was mostly distributed among the participants with tertiary education level. The knowledge score from this study is fairly high compared to that among healthcare workers in Pakistan [8] which was of 35.2% regarding the use of face masks to limit the spread of COVID- 19. High education level and being married are signs of responsibility and can explain the fact that these categories of people were more knowledgeable than others in our study as was found in several other studies on KAP towards COVID-19 [20, 21].

In our study, the attitude to face masks differed across age group, education level, occupation, marital status and residence of participants similar to findings by Hager et al [20]. Although, only 69.4% (773/1114) respondents agreed being confident enough to correctly put on a face mask, 83.4% believed that a face mask can protect against COVID-19 and 75.9% disagreed to having ever shared their face mask. The findings differed from similar studies in Pakistan and in Hong Kong about the use of face mask where by 88.5% and 88.4% of participants respectively, reported being confident enough to know the correct steps of wearing a face mask [8, 12]. Ho [22] in Hong Kong found that 52.0% of participants reported knowing the correct procedure for wearing a face mask to protect themselves from getting infected during the Influenza H1N1epidemic. In our study 83.4% participants believed that a face mask can protect against COVID-19. This was slightly higher than the findings by Azlan et al [13] who found that 76.7% of participants in Malaysia believed that a face mask can protect against COVID-19. However, our findings were similar to those in Hong Kong during the influenza H1N1 epidemic where 88.5% participants believed that wearing a face mask is a good way to protect against Influenza-like illness [22].

The participants (24.1%) who agreed sharing their face mask in this study constitute a gap for which the government should further sensitize the community to avoid the spread of the COVID-19 in the country.

The practice of wearing a face mask in the public place was reported to be better (95.2%) than what was found in Hong Kong [22], Malaysia [13] and Ghana [20] where they reported a lower use of face masks (69.2%, 51.2% and 32.3%) respectively. The findings of this study were similar to the studies done in Uganda by [14, 15] and Pakistan by [8] where they found that 99.26%, 93% and 93.9% of participants respectively, reported using face mask to protect themselves against COVID-19. To wash hands before and after wearing a face mask was not reported to be done among 39.7% of participants and this compares better than the study done by Lee et al [12] in Hong Kong where the majority of the participants did not perform hand hygiene before putting on (91.5%), taking off (97.3%) or after disposing of (91.5%) the face mask. The findings from our study may be explained by the fact that Ugandans have faced more epidemics such Ebola and Marburg [23, 24] and already could be more sensitized, indicating a good infection preventive control level. However, 51.5% of participants in this study reported removing the face mask if there was a need to talk to someone. This finding differs from the study in Pakistan where 13.8% of participants reported that they can remove their face mask while talking to someone [8]. This difference could be due to the fact that only health workers considered to be more knowledgeable, were considered in the study done in Pakistan.

The researchers were unable to incorporate qualitative methods of research such as focus group discissions and in-depth due to the limitations on gatherings although such approaches would have enriched our findings. The study tool had only two languages—Runyakitara which is widely used in western Uganda and English. This posed a limitation for some participants and in such cases a third language–Luganda, more widely spoken in the central region was used by the researcher. This required reverse translation by a language expert and training of the researchers. As the study was done in western region, the findings from this study may not be generalizable for the whole country.

## 5. Conclusion

In summary, most of western Ugandans had satisfactory knowledgeable, a positive attitude, and observed good practices towards the use of face mask to limit the spread of COVID-19 in the community. Despite these findings, there is still much more to be done in terms of knowledge, attitude and practices among western Ugandans to reach the hundred percent awareness needed as a way of infection disease control. The government of Uganda could use the educated people to help in mobilization of the community on the proper steps of wearing and disposing off a face mask to limit the spread of COVID-19 in western Uganda and the rest of the country.
